# Overcoming the Neonatal Limitations of Inducing Germinal Centers through Liposome-Based Adjuvants Including C-Type Lectin Agonists Trehalose Dibehenate or Curdlan

**DOI:** 10.3389/fimmu.2018.00381

**Published:** 2018-02-28

**Authors:** Maria Vono, Christiane Sigrid Eberhardt, Elodie Mohr, Floriane Auderset, Dennis Christensen, Mirco Schmolke, Rhea Coler, Andreas Meinke, Peter Andersen, Paul-Henri Lambert, Beatris Mastelic-Gavillet, Claire-Anne Siegrist

**Affiliations:** ^1^WHO Collaborative Center for Vaccine Immunology, Department of Pathology-Immunology, University of Geneva, Geneva, Switzerland; ^2^WHO Collaborative Center for Vaccine Immunology, Department of Pediatrics, University of Geneva, Geneva, Switzerland; ^3^Vaccine Adjuvant Research, Department of Infectious Disease Immunology, Statens Serum Institut, Copenhagen, Denmark; ^4^Department of Microbiology and Molecular Medicine, University of Geneva, Geneva, Switzerland; ^5^Infectious Disease Research Institute, Seattle, WA, United States; ^6^Valneva Austria GmbH, Vienna, Austria

**Keywords:** T follicular helper cells, germinal centers, neonates, vaccines, adjuvants

## Abstract

Neonates and infants are more vulnerable to infections and show reduced responses to vaccination. Consequently, repeated immunizations are required to induce protection and early life vaccines against major pathogens such as influenza are yet unavailable. Formulating antigens with potent adjuvants, including immunostimulators and delivery systems, is a demonstrated approach to enhance vaccine efficacy. Yet, adjuvants effective in adults may not meet the specific requirements for activating the early life immune system. Here, we assessed the neonatal adjuvanticity of three novel adjuvants including TLR4 (glucopyranosyl lipid adjuvant-squalene emulsion), TLR9 (IC31^®^), and Mincle (CAF01) agonists, which all induce germinal centers (GCs) and potent antibody responses to influenza hemagglutinin (HA) in adult mice. In neonates, a single dose of HA formulated into each adjuvant induced T follicular helper (T_FH_) cells. However, only HA/CAF01 elicited significantly higher and sustained antibody responses, engaging neonatal B cells to differentiate into GCs already after a single dose. Although antibody titers remained lower than in adults, HA-specific responses induced by a single neonatal dose of HA/CAF01 were sufficient to confer protection against influenza viral challenge. Postulating that the neonatal adjuvanticity of CAF01 may result from the functionality of the C-type lectin receptor (CLR) Mincle in early life we asked whether other C-type lectin agonists would show a similar neonatal adjuvanticity. Replacing the Mincle agonist trehalose 6,6′-dibehenate by Curdlan, which binds to Dectin-1, enhanced antibody responses through the induction of similar levels of T_FH_, GCs and bone marrow high-affinity plasma cells. Thus, specific requirements of early life B cells may already be met after a single vaccine dose using CLR-activating agonists, identified here as promising B cell immunostimulators for early life vaccines when included into cationic liposomes.

## Introduction

Neonates and young infants are particularly vulnerable to infectious diseases and providing protection at that early time in life remains challenging (1). One example is influenza, against which currently available vaccines elicit weak responses. Newborn and infant protection against influenza may currently only be achieved by maternal immunization and transplacental transfer of maternal antibodies to the fetus. However, maternal antibodies wane rapidly after birth. Between 6 and 25 months of life, trivalent influenza vaccines (TIV) have limited immunogenicity and protective efficacy (2, 3), which may be enhanced in part by MF59^®^ adjuvantation (4). In contrast, influenza vaccines for infants younger than 6 months are lacking: TIV showed poor efficacy (3) and the live attenuated intranasal vaccine appeared too reactogenic in this age group (5). MF59^®^-adjuvanted vaccines have not yet been tested in young infants. In infant mice, MF59^®^ induced adult-like antibody titers, T follicular helper (T_FH_) cells, germinal centers (GCs) and protection against influenza challenge but failed to do so in neonatal mice (6), indicating the existence of different immunological requirements in newborns.

The mechanisms underlying the limitations of early life B cell responses are multiple and not well understood yet. Preclinical murine models suggest that the pattern of early life antibody responses, hallmarked by low antibody titers with limited persistence, reflects the restricted induction of GCs-derived B cells (1, 7). So far, only one adjuvant, LT-K63, was shown to enhance the GC reaction and antibody responses in neonatal mice (8) but its clinical development has been stopped due to transient adverse reactions in humans and its mechanisms of action remain unknown. We and others have previously identified a critical role for T_FH_ cells in the impaired development of GC reactions following neonatal immunization with the current aluminum-containing vaccines (9, 10). Hence, new adjuvants targeting these specific neonatal requirements are needed.

Several novel candidate adjuvants in advanced clinical development are being assessed within the Advanced Immunization Technologies (ADITEC) collaborative research program (11). Within this consortium, we initially selected three promising adjuvants to explore their neonatal adjuvanticity. Glucopyranosyl lipid adjuvant (GLA)-squalene emulsion (SE) is a SE combined with the TLR4 agonist GLA. In adult mice, GLA-SE elicited potent T_H_1 responses and protective antibody titers to influenza (12, 13). The induction of strong antibody responses in adults was confirmed in a human phase 1 trial (14). IC31^®^ consists of the cationic membrane interacting peptide KLK (KLKL_5_KLK) and of a single-stranded DNA-phosphodiester oligo-d(IC)13 (ODN1a), a TLR9 agonist. IC31^®^ induced strong T_H_1, and substantial murine B cell responses in adult mice (15) and improved influenza vaccine responses in adult and aged mice (16). An IC31^®^-containing tuberculosis (TB) vaccine was shown to induce potent T_H_1 responses in humans (17). In neonatal mice, IC31^®^-containing vaccines elicited adult-like T_H_1 responses to TB antigens (18, 19) and enhanced T_H_1 responses, antibody responses, and protection against pneumococcal challenge (20). The combined T_H_1-driving and B cell supporting functions of GLA-SE and IC31^®^ could thus potentially address some key requirements for neonatal adjuvantation.

CAF01 is an adjuvant composed of a liposomal delivery vehicle formed by the cationic surfactant dimethyldioctadecyl-ammonium (DDA) incorporating the immunostimulator trehalose 6,6′-dibehenate (TDB) (21). CAF01 signals *via* the C-type lectin receptor (CLR) Mincle, activating the Syk/Card9 pathway to increase the production of pro-inflammatory cytokines (22, 23). In adult mice, CAF01 elicited strong T_H_1/T_H_17 responses but moderate antibody responses to influenza hemagglutinin (HA) (12). In neonates, CAF01 elicited mixed T_H_1/T_H_17 responses against TB antigens (24). Its neonatal B cell adjuvanticity had not yet been assessed.

Here, we used these three novel adjuvant formulations to explore the capacity of the neonatal and adult murine immune system to elicit GC B cell responses to influenza HA. Our findings identified for the first time CAF01 as a potent neonatal adjuvant able to strongly enhance neonatal B cell responses and thus the protective efficacy of early life vaccines. Interestingly, formulating Curdlan, a different CLR agonist, in DDA similarly increased primary neonatal B cell responses to HA, revealing the great potential of CLR agonists as B cell adjuvants for early life vaccines.

## Materials and Methods

### Mice

Adult CB6F1/OlaHsd females were purchased from Harlan (Horst, The Netherlands) together with BALB/c OlaHsd females and C57BL/6 OlaHsd males. The latter were crossed to produce F1 CB6F1 mice. All mice were bred, kept in pathogen-free animal facilities in accordance with local guidelines and used at 1 week (neonates) or 6–8 weeks (adults) of age. All animal experiments were approved by the Geneva veterinary office and conducted under relevant Swiss and European guidelines.

### Antigens, Adjuvants, and Immunization

We used an experimental monovalent purified subunit influenza vaccine composed of HA from the influenza strain H1N1 A/California/7/2009 [Novartis Vaccines (a GSK company), Siena, Italy]. Groups of 5–8 CB6F1 neonatal (1-week-old) and adult mice were immunized subcutaneously (s.c.) with 100 µl of the plain HA (1 µg) or in combination with either CAF01 (250 µg DDA/50 μg TDB, Statens Serum Institut, Copenhagen, Denmark), IC31^®^ (KLK/ODN1a = 100 nmol/4 nmol, Valneva Austria GmbH), GLA-SE (5 µg GLA and 2% v/v squalene, Infectious Diseases Research Institute, Seattle, WA, USA), or DDA-Curdlan (250 µg DDA/50 μg Curdlan, Statens Serum Institut, Copenhagen, Denmark) produced according to the protocol previously described for DDA-TDB (25). Curdlan was purchased from Sigma-Aldrich.

Mice were immunized at the base of the tail and inguinal draining lymph nodes (LNs) were harvested, except for the experiments with GLA-SE in which mice from both age groups were injected s.c. (100 µl) at the scruff of the neck and brachial draining LNs harvested. This use of the base of the tail as injection site was required to comply with the new local animal welfare guidance to limit procedures requiring anesthesia. We carefully checked that this change did not affect our results (Figure S1 in Supplementary Material).

### Influenza Challenge

Viral challenge was performed as recently described (6) using a mouse-adapted H1N1 Influenza strain (A/Netherlands/602/2009, passage 2 in mice). Virus was grown on MDCK cells (ATCC). Fifty-six days post-immunization, 2 × 10^3^ PFU of virus in 20 µl sterile PBS were instilled intranasally under anesthesia induced by Ketasol^®^ (Graeub) and Rompun^®^ (Bayer). Mice were observed daily to monitor body weight and survival. Mice showing more than 20% of body weight loss were humanely euthanized.

### Enzyme-Linked Immunosorbent Assay (ELISA) and Avidity of HA-Specific Antibodies

Mice were bled from the tail vein at the indicated time points except for neonatal mice at day 0 that were bled by decapitation. Titration of HA-specific total IgG, IgG1, and IgG2a titers was performed by ELISA on individual serum samples as previously described (6).

Avidity was measured by ELISA as the overall strength of binding between antibody and antigen, using plates incubated for 15 min with increasing concentration of ammonium thiocyanate (NH_4_SCN) from 0 to 1.5 M. Antibody avidity was defined as the amount of antibody eluted for each increment of NH_4_SCN concentration. Arbitrarily, the percentage of antibody eluted between 0 and 0.25 M of NH_4_SCN was assigned as low-avidity antibody fraction, between 0.25 and 0.75 M as intermediate and >0.75 M as high-avidity antibody fraction.

### Enzyme-Linked Immunospot (ELISpot) Assay for HA-Specific Plasma Cells

Hemagglutinin-specific plasma cells were quantified by direct *ex vivo* ELISpot assay as previously described (6).

### Flow Cytometric Analysis

Cells from the two draining LNs of each individual mouse were pooled and stained with fluorescently labeled antibodies to GL7, CD8, B220, TCR-β, CD95 (Fas) (all from BD Biosciences), PD-1, Ter119, GR1, CD11c (all from eBioscience), and CD4 (all from BioLegend). CXCR5 staining was performed using purified anti-CXCR5 (BD Biosciences), followed by FITC anti-rat IgG (Southern Biotech), and normal rat serum (eBioscience). The stained cells were analyzed using a Gallios cytometer (Beckman Coulter) and the generated data analyzed using FlowJo Software (Tree Star).

### Immunohistochemistry

Germinal centers in the draining LNs of immunized mice were stained and quantified as previously described (6). Sections were visualized and photographed with a Zeiss LSM700 confocal microscope (objective: 20×) or a Mirax scan microscope (Zeiss). Images were acquired with Zeiss LSM image browser software (Zeiss) or the Pannoramic Viewer software (3DHistec).

### Statistical Analysis

Data were analyzed using Prism 6.0 (GraphPad Software) and presented as mean ± SEM of at least three independent experiments. Difference between groups was analyzed as described in figure legends. *P*-values less than 0.05 were considered statistically significant.

## Results

### Novel Adjuvants Exert Distinct Effects on B Cell Responses to Vaccination in Adult and Early Life

**Figure 1:**
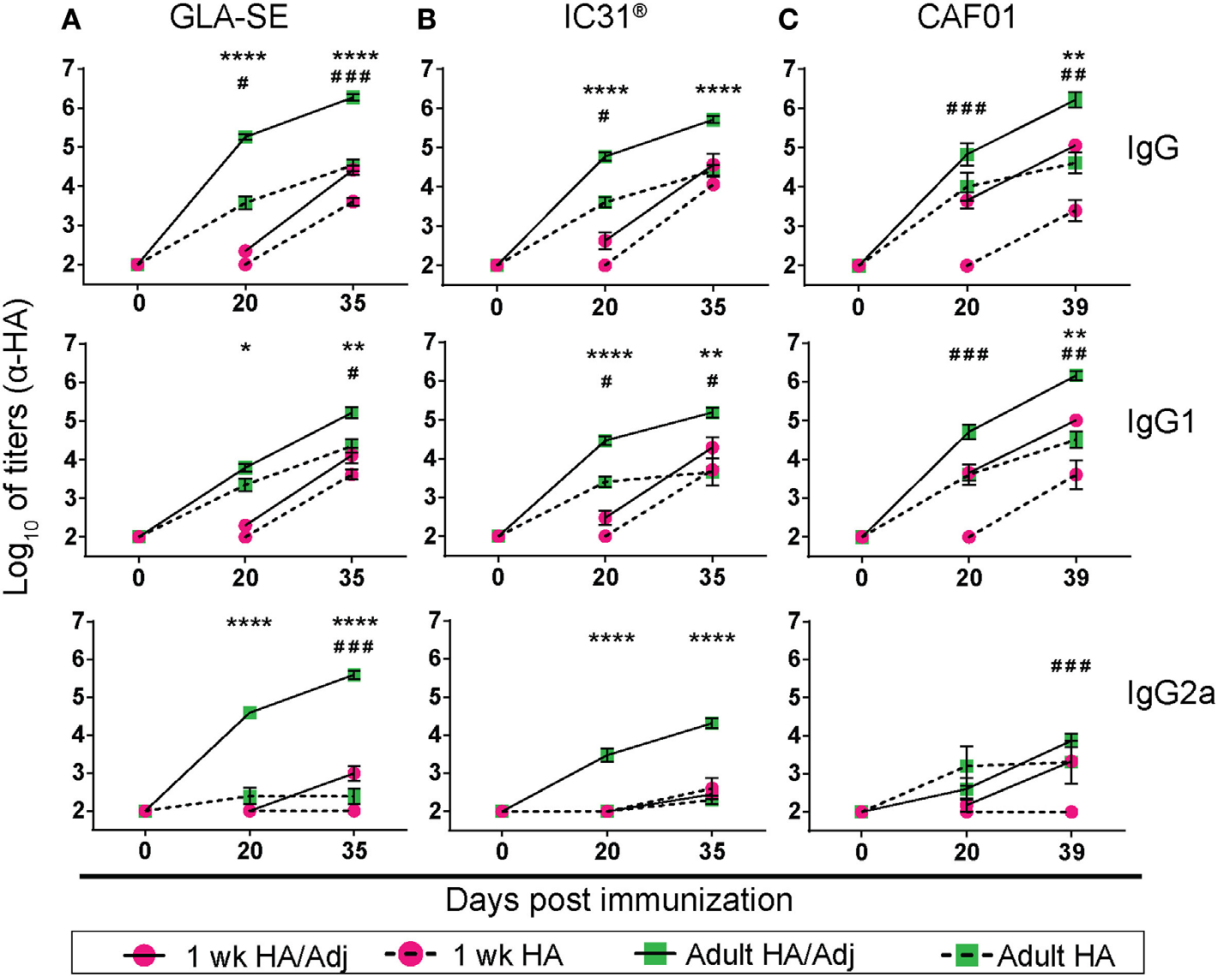
CAF01 adjuvantation strongly enhances primary hemagglutinin (HA)-specific antibody responses in neonates. Neonatal (1 week) and adult CB6F1 mice were immunized subcutaneously at days 0 and 21 with HA alone or formulated in combination with an adjuvant (Adj): glucopyranosyl lipid adjuvant (GLA)-squalene emulsion (SE) **(A)**, IC31^®^ **(B)**, or CAF01 **(C)**. Sera were drawn before (day 0), 3 weeks post-prime (day 20), and post-boost (day 35 or 39) and total HA-specific IgG, IgG1, and IgG2a antibody titers were measured by enzyme-linked immunosorbent assay. Neonatal mouse serum at day 0 was obtained by decapitation of different naïve newborns. Values represent mean logarithmic titers (log 10) of five to eight mice per group ± SEM. Adjuvanted groups were compared with the corresponding non-adjuvanted groups using the Mann–Whitney *U* test. *HA/Adj vs HA statistics in adult mice: *P < 0.05, **P < 0.01, ***P < 0.001, ****P < 0.0001. ^#^HA/Adj vs HA statistics in neonatal mice: ^#^*P* < 0.05, ^##^*P* < 0.01, ^###^*P* < 0.001.

We first compared the antibody titers elicited by a subunit monovalent influenza vaccine containing HA administered alone or formulated with GLA-SE, IC31^®^, or CAF01. Neonates (referred to as “1 week”) and adult mice were immunized twice at days 0 and 21. In adults, a single dose of unadjuvanted HA was sufficient to elicit HA-specific antibody responses (Figure 1). These responses were strongly enhanced by each tested adjuvant (Figure 1), as recently reported (12). The highest primary IgG responses were induced by HA/GLA-SE (Figure 1A), followed by HA/IC31^®^ (Figure 1B), and HA/CAF01 (Figure 1C). HA/GLA-SE induced antibodies with a more pronounced IgG2a profile, while HA/IC31^®^ and HA/CAF01 primarily induced IgG1 antibodies.

Consistently with our previous findings (6), neonates did not raise detectable primary IgG responses to unadjuvanted HA. They also poorly responded to a single dose of HA/GLA-SE or HA/IC31^®^. In contrast, HA/CAF01 strongly increased primary HA-specific IgG and particularly IgG1 responses (Figure 1C). Neonatal responses to HA/CAF01 were significantly higher than to HA/IC31^®^ (*P* ≤ 0.004) and HA/GLA-SE (*P* ≤ 0.0004). Neonatal responses to the second dose of HA/CAF01 were also significantly higher than those elicited by unadjuvanted HA, although neither primary nor secondary responses reached the titers elicited by adjuvanted vaccines in adults (Figure 1).

Thus, adult and neonatal requirements for B cell adjuvanticity differ: HA/GLA-SE is the strongest antibody inducer in adults, whereas only CAF01 adjuvantation succeeds in eliciting potent primary responses in neonates.

### The Three Adjuvants Successfully Induce T Follicular Helper Cells in Neonates

**Figure 2:**
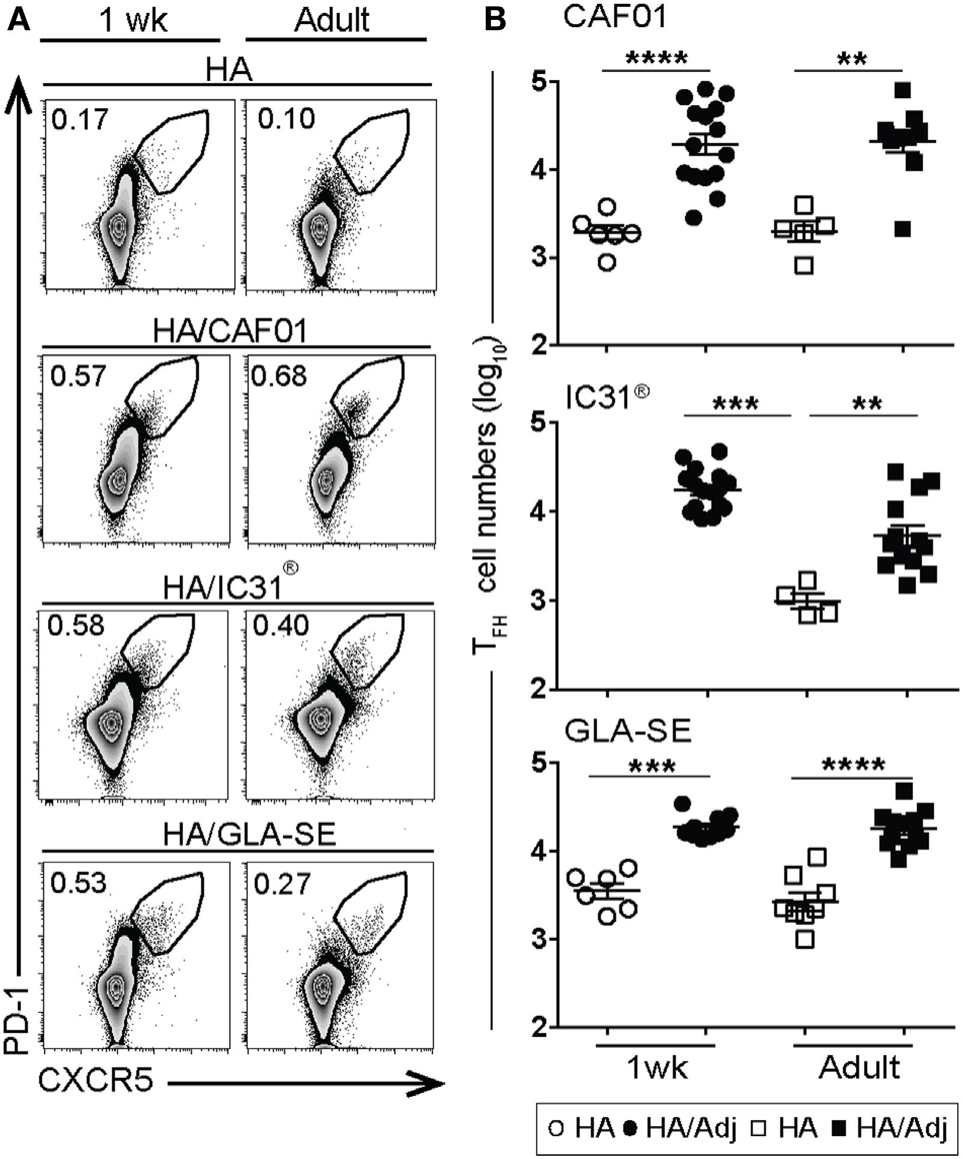
Induction of T follicular helper cells in draining lymph nodes (LNs) by vaccine adjuvants. 1-week-old and adult CB6F1 mice were immunized subcutaneously with hemagglutinin (HA) unadjuvanted or in combination with either glucopyranosyl lipid adjuvant (GLA)-squalene emulsion (SE), IC31^®^, or CAF01 and, respectively, 10 (experiments with GLA-SE) or 12 days post-vaccination the draining LNs were harvested to measure CXCR5^high^ PD-1^high^ T_FH_ cells by flow cytometry. **(A)** Representative dot plots show CXCR5^high^ PD-1^high^ T_FH_ cells among the CD4^+^ T cells and **(B)** graphs report the total number of T_FH_ cells. Dots show values per individual mouse (*n* ≥ 5 per group) whereas black bars indicate means ± SEM. Data pool of at least two independent experiments. Mann–Whitney *U* test: **P < 0.01, ***P < 0.001, ****P < 0.0001.

T_FH_ cells are critical for GC formation, controlling the number of GC B cell divisions, and are thus essential for the generation of high-affinity matured antibodies (26, 27). The induction of T_FH_ cells in early life is challenging: we previously reported that both alum-adsorbed (10) and MF59^®^-adjuvanted (6) vaccines failed to generate T_FH_ cells in neonates. CD4^+^ CXCR5^high^PD-1^high^ T_FH_ cells were thus measured in the draining LNs after neonatal or adult (control) immunization. T_FH_ cell responses increased significantly upon HA/CAF01 immunization in both age groups, reaching similar numbers in adults and neonates (Figures 2A,B).

Unexpectedly, despite the poor neonatal primary antibody responses to HA/GLA-SE and HA/IC31^®^, a significant increase in CD4^+^CXCR5^high^PD-1^high^ T_FH_ cells, reaching adult levels, was also observed in HA/GLA-SE- and HA/IC31^®^-immunized neonates (Figures 2A,B). Neonatal T_FH_ cell responses elicited by HA/GLA-SE peaked at day 10 but dropped down relatively rapidly over time (Figure S2A in Supplementary Material). In contrast, less than 0.05% of CD4^+^ CXCR5^high^PD-1^high^ T_FH_ cells were repeatedly observed in naïve infant mice (not shown). Thus, all tested adjuvants passed the challenge of inducing T_FH_ cells in neonates.

### Only HA/CAF01 Elicits *Bona Fide* GC Responses in Neonates

**Figure 3:**
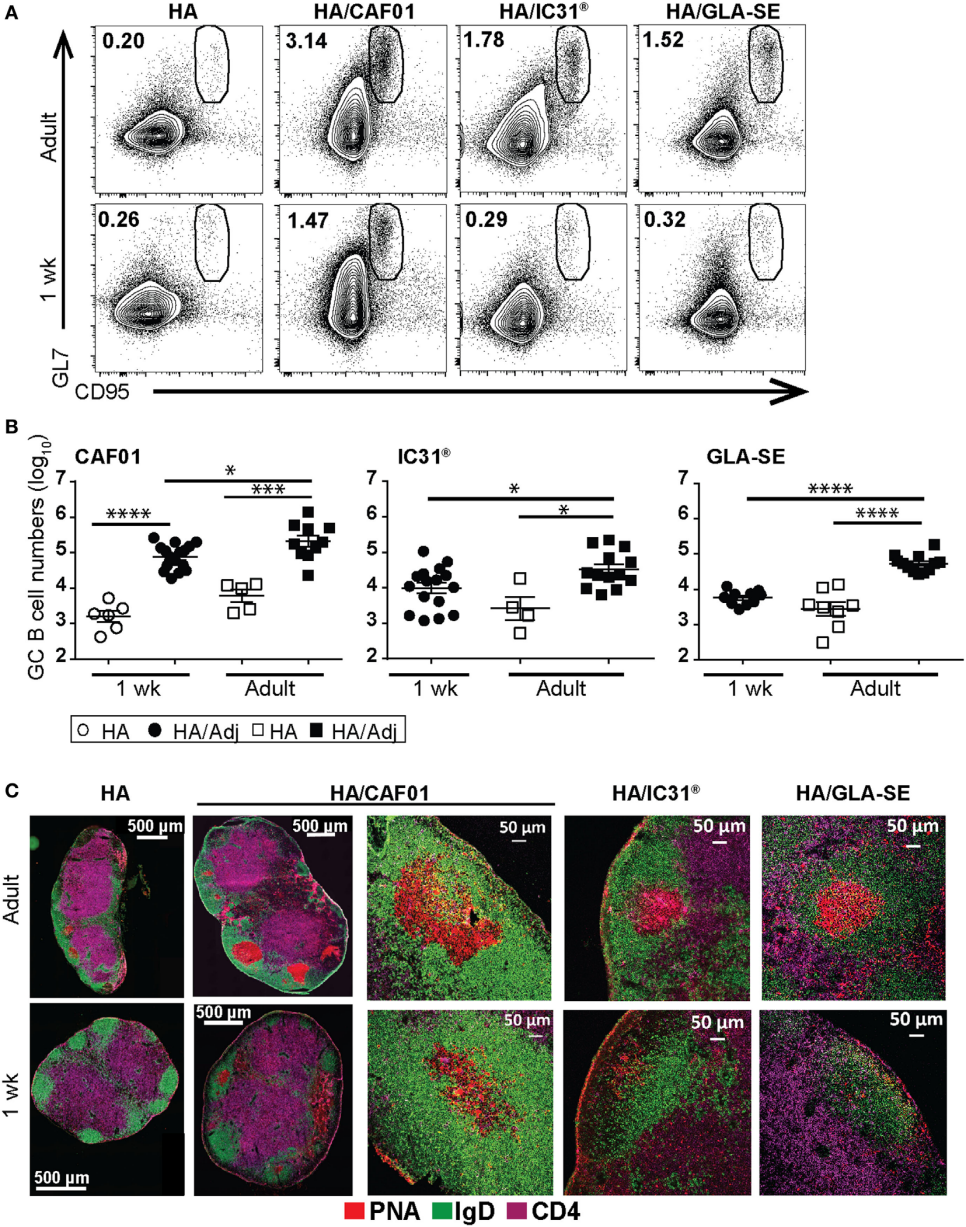
Neonatal primary antibody responses elicited by hemagglutinin (HA)/CAF01 correlate with the induction of *bona fide* germinal centers (GCs). Draining lymph nodes (LNs) were harvested 10 [experiments with glucopyranosyl lipid adjuvant (GLA)-squalene emulsion (SE)] or 12 days (experiments with IC31^®^ or CAF01) after immunization of adult and neonatal mice. **(A,B)** Samples were analyzed by flow cytometry to quantify the frequency and the total numbers of GL7^+^CD95^+^ GC B cells. **(A)** Representative dot plots show the frequency of GL7^+^CD95^+^ GC B cells among the B220^+^ B cells for all the indicated conditions; **(B)** graphs report the total numbers of GC B cells induced by the distinct adjuvants. In neonates, total B220^+^ B cell numbers increased with adjuvantation but were comparable between groups (not shown). Dots show values per individual mouse (*n* ≥ 4 per group) whereas black bars indicate mean ± SEM. Data pool of at least two independent experiments. Mann–Whitney *U* test: *P < 0.05, ***P < 0.001, ****P < 0.0001. **(C)** Representative sections of adult and neonatal draining LNs showing immunohistochemical staining for IgD (green), the GC marker peanut agglutinin (PNA; red) and CD4 (purple); GC B cells are IgD^−^PNA^+^. Five to eight sections per mouse from six to eight mice/group were analyzed.

As GC reactions are essential determinants of the magnitude and the quality of antibody responses and humoral memory, we next assessed whether these adjuvants differed in their capacity to induce GCs. B220^+^GL7^+^CD95^+^ GC B cells were quantified by flow cytometry in draining LNs at day 10 or 12 upon immunization, as indicated in figure legends. In adults, the three adjuvants significantly increased the numbers of GC B cells when compared with unadjuvanted HA (Figures 3A,B). The few GC B cells in the draining LNs of neonates that received unadjuvanted HA were comparable with that observed in non-draining LNs (not shown), representing background levels. In neonates, adjuvantation with CAF01 strongly augmented both the number (Figure 3B) and proportion of GC B cells compared with unadjuvanted HA (HA/CAF01: 1.99% ± 0.22 vs HA: 0.30% ± 0.05, *P* < 0.0001). In contrast, only few GC B cells were observed following HA/IC31^®^ or HA/GLA-SE neonatal immunization. Neonatal GC B cell responses induced by HA/GLA-SE remained low even later after immunization (Figure S2B in Supplementary Material). In contrast, in adult mice higher GC B cell responses were observed at day 12 following vaccination with HA/GLA-SE (Figure S2B in Supplementary Material). Thus, a single dose of HA/CAF01-induced significantly higher numbers of neonatal GC B cells than unadjuvanted HA, HA/GLA-SE, or HA/IC31^®^, in consistency with the observed higher antibody titers (Figure 1).

To explore whether the GC B cells identified by flow cytometry were organized in *bona fide* GC structures, we imaged follicular B cells (IgD, green), GC B cells [peanut agglutinin (PNA), red] and CD4^+^ T cells (CD4, purple) in draining LN sections post-prime (Figure 3C). In adults, highly organized IgD^−^PNA^+^ GC structures were observed. These GCs were very few in adult mice that received unadjuvanted HA and increased following immunization with the adjuvanted HA, irrespective of which adjuvant (Figure 3C and data not shown). Vaccination of neonatal mice with unadjuvanted HA, HA/IC31^®^, or HA/GLA-SE did not generate *bona fide* GC structures. In contrast, well-organized GCs were observed in HA/CAF01-immunized neonates (Figure 3C), albeit PNA^+^ GCs induced by HA/CAF01 remained fewer (1 week: 1.85 ± 0.29 vs adults: 3.44 ± 0.46, *P* ≤ 0.01 per LN section) and smaller (1 week: 23,883 ± 9,973 µm^2^ vs adult: 42,888 ± 9,810 µm^2^) than in adults. Thus, a single dose of HA/CAF01 elicited *bona fide* GCs even in neonates.

### A Single Dose of HA/CAF01 Elicits High-Affinity Sustained B Cell Responses

**Figure 4:**
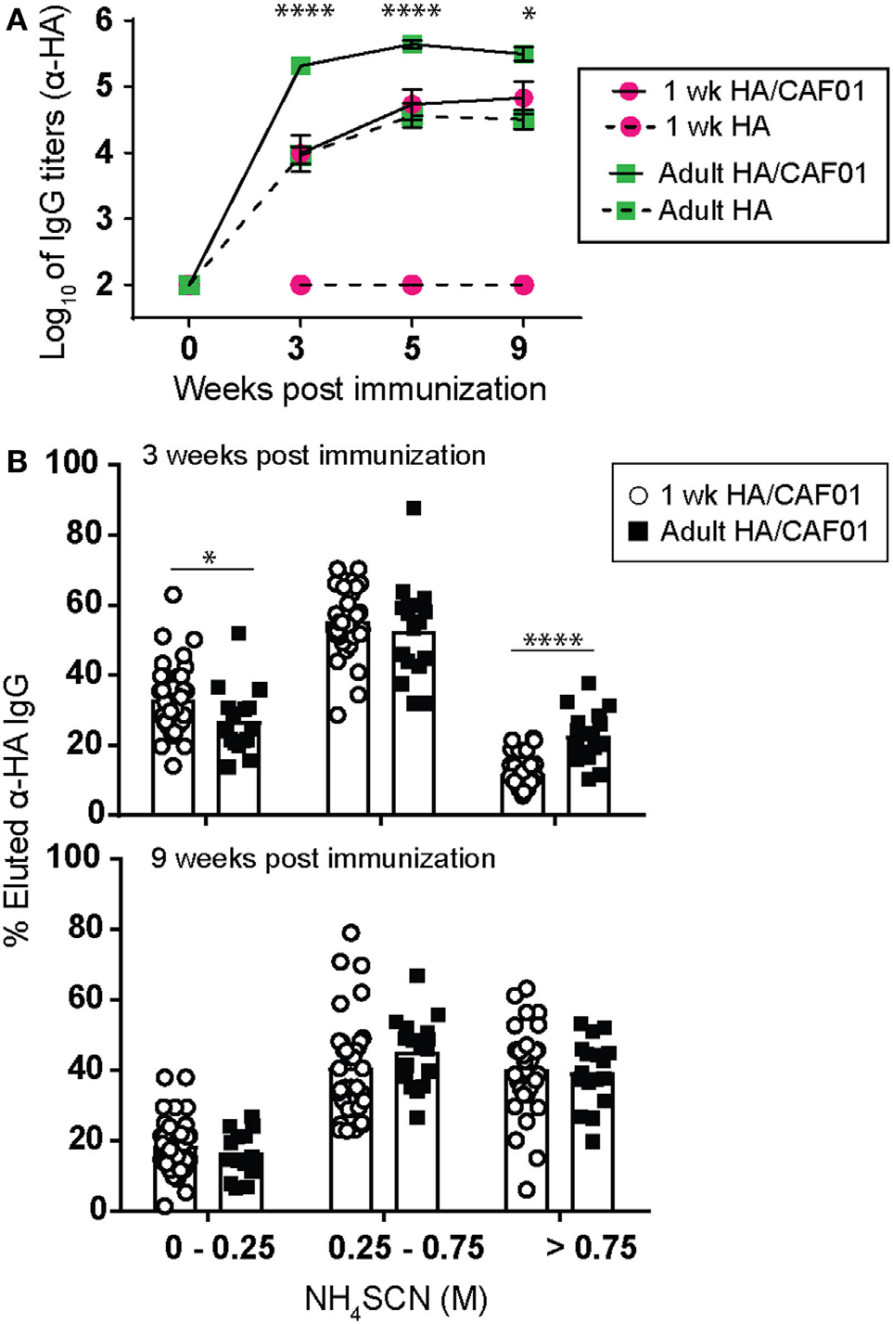
A single dose of hemagglutinin (HA)/CAF01 elicits sustained B cell responses. 1-week-old and adult CB6F1 mice were immunized subcutaneously (s.c.) at day 0 with HA/CAF01 or HA. **(A)** Sera samples were drawn at the indicated time points to measure HA-specific IgG titers. Values represent mean logarithmic titers (log 10) of at least six mice per group ± SEM. HA/CAF01 significantly increased antibody titers when compared with unadjuvanted HA in both age groups at all time points after day 0 (statistics not shown). HA-specific antibody titers elicited by HA/CAF01 in neonates remained significantly lower than in adults: Mann–Whitney *U* test: *P < 0.05, ****P < 0.0001. **(B)** The avidity of adult and neonatal HA-specific IgG antibodies early (3 weeks) and late (9 weeks) post-immunization is shown as percentages of antibodies eluted at different concentrations of ammonium thiocyanate (NH_4_SCN). Arbitrarily, the antibody fraction eluted between 0 and 0.25 M of NH_4_SCN was assigned as low-avidity antibody fraction, 0.25–0.75 M as intermediate and >0.75 M as high-avidity antibody fraction. Dots show values per individual mouse (*n* ≥ 6 per group) whereas black bars indicate means. Data pool of at least three independent experiments. Mann–Whitney *U* test: *P < 0.05, ****P < 0.0001.

Early life immunization in general induces reduced titers of antibodies and these are of lower avidity compared with responses achieved by immunization in adults (1, 28). We thus asked whether a single dose of HA/CAF01 also affected these hallmarks of neonatal B cell responses. First, serum IgG antibody responses were measured for up to 9 weeks post-prime. In adults, HA/CAF01-induced significantly higher antibodies than unadjuvanted HA and these responses reached a plateau already 3 weeks after prime (Figure 4A). A similar enhancement of antibody responses was observed in neonates, although with slower kinetics (peak at 5 weeks, Figure 4A) and, as previously reported, lower magnitude (Figure 1C). HA/CAF01-induced antibodies persisted for at least 9 weeks post-prime (last time point assessed, Figure 4A) in both neonates and adults.

CAF01 (29) and IC31^®^ (15) both induce a depot effect which may allow a slow antigen release from the injection site and contribute to sustained antibody responses. However, antibodies induced by HA/IC31^®^ in neonates remained very low over time (Figure S3 in Supplementary Material). Thus, a depot effect is not sufficient to eventually elicit antibody responses in early life.

The avidity of HA-specific IgG antibodies was measured early (3 weeks) and late (9 weeks) after a single dose of HA/CAF01 (Figure 4B). Avidity is shown as percentages of eluted HA-specific antibodies after treatment with increasing concentrations of ammonium thiocyanate (NH_4_SCN) (30). At 3 weeks post-prime, a higher proportion of low-avidity antibodies (eluted with a low concentration of ammonium thiocyanate) was observed in neonates than in adults (Figure 4B). However, avidity increased rapidly following neonatal immunization with HA/CAF01, reaching adult-like levels at 9 weeks (Figure 4B).

We previously reported that neonatal immunization with aluminum adjuvants elicits abortive and rapidly terminated GC responses (10). In contrast, a single dose of HA/CAF01-induced sustained GC activity: HA/CAF01-induced GC B cells persisted in both age groups for at least 5 weeks after a single immunization, albeit at significantly lower numbers than in adults in agreement with previous results (Figure S4 in Supplementary Material). Thus, the neonatal B cell adjuvanticity of CAF01 results into a potent and sustained induction of *bona fide* GC B cells, which rapidly generates high-affinity and persistent primary antibody responses.

### A Single Dose of HA/CAF01 Protects Mice against Influenza Virus Challenge

**Figure 5:**
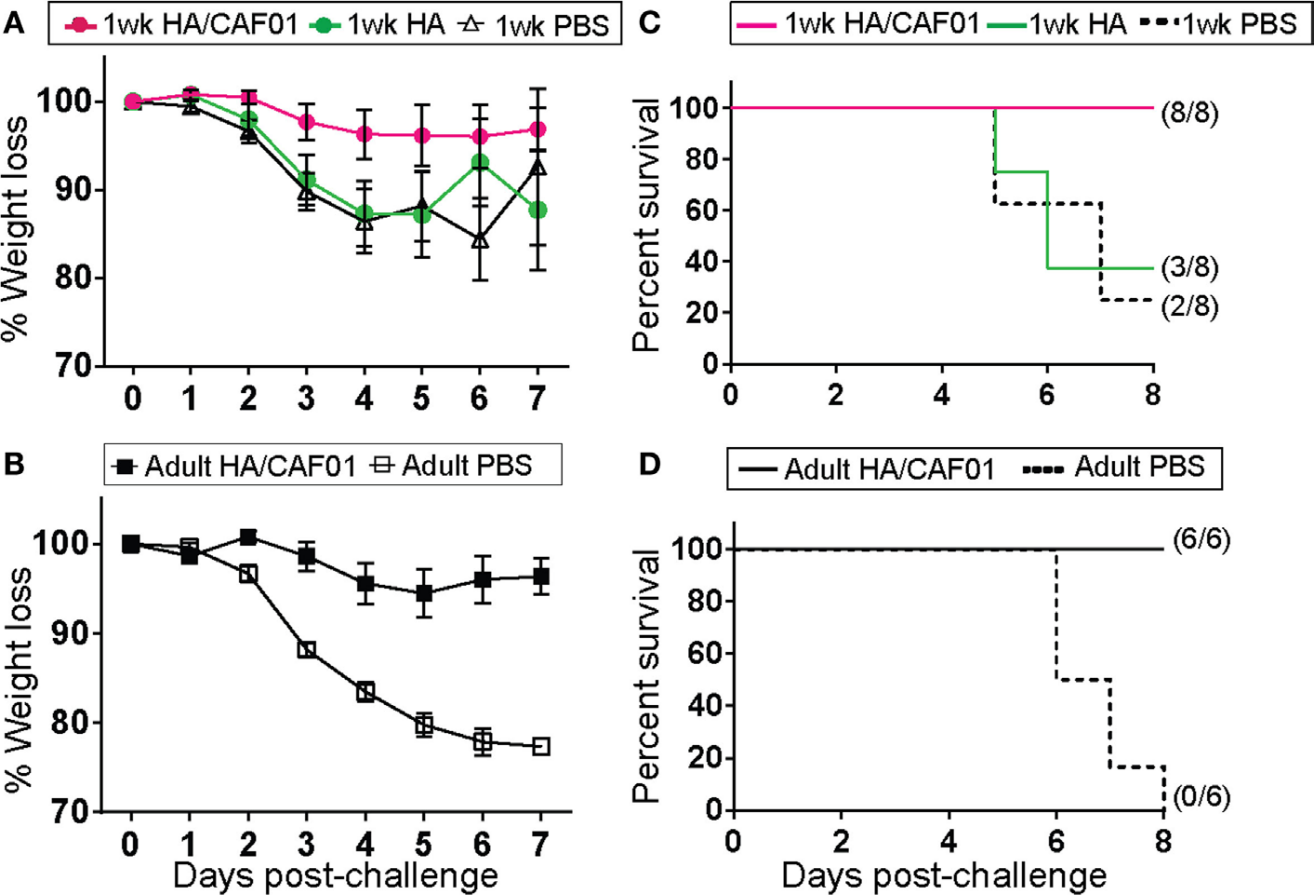
A single dose of hemagglutinin (HA)/CAF01 protects against influenza virus challenge. 1-week-old and adult CB6F1 mice were immunized subcutaneously with either unadjuvanted HA, HA/CAF01, or PBS as control. 56 days after injection, 2 × 10^3^ PFU of mouse-adapted A/Netherlands/602/09 (H1N1) influenza virus were instilled intranasally under anesthesia. Mice were observed daily up to day 8 post-challenge. **(A,B)** The daily weights of each animal were calculated compared with their respective weight on the day of challenge, and data are shown as the average percentage of initial weight for each group. **(C,D)** Graphs show survival rates post-challenge. Data are representative of two independent experiments.

We next asked whether the HA-specific antibody responses elicited by a single dose of HA/CAF01 in neonates were sufficient to confer protection against influenza viral challenge, which is difficult to achieve. Neonatal mice received a single dose of unadjuvanted HA, HA/CAF01, or PBS (control) and were challenged intranasally 8 weeks later with a lethal dose of matching influenza A/H1N1 virus. As additional controls, we challenged HA/CAF01- (positive control) or PBS- (negative control) injected adult mice. The body weight of mice that received PBS or unadjuvanted HA declined to 80% on average within 6 days (Figures 5A,B). In contrast, the body weight of HA/CAF01-immunized mice only transiently declined by less than 10% (4–6%) in both age groups (Figures 5A,B). The protection mediated by HA/CAF01 translated into a survival rate of 100% in both age groups (Figures 5C,D). In neonates, CAF01 adjuvantation was required for protection, as only 3/8 mice that received unadjuvanted HA survived up to day 8 (Figure 5C). Among these survivors, two mice had signs of severe infection and lost considerable body weight (17.1 and 19.9% of weight loss, respectively). Similarly, in the group of neonates that received PBS, one of two surviving mice had signs of severe infection on day 8 (18.3% of weight loss). Only one mouse in each group either was not fully infected or recovered spontaneously. Thus, a single dose of HA/CAF01 is sufficient to confer protection against influenza viral challenge even when given to neonates.

### Primary Responses to HA/DDA-Curdlan

The potent neonatal B cell adjuvanticity of the Mincle-activating CAF01 adjuvant suggests that CLR targeting may be critical in neonates rather than signaling through TLRs. To explore this possibility, we tested the neonatal B cell adjuvanticity of Curdlan, which binds the CLR Dectin-1. Thus, by replacing the CAF01 immunostimulator TDB with Curdlan in the cationic surfactant DDA, we generated DDA-Curdlan.

**Figure 6:**
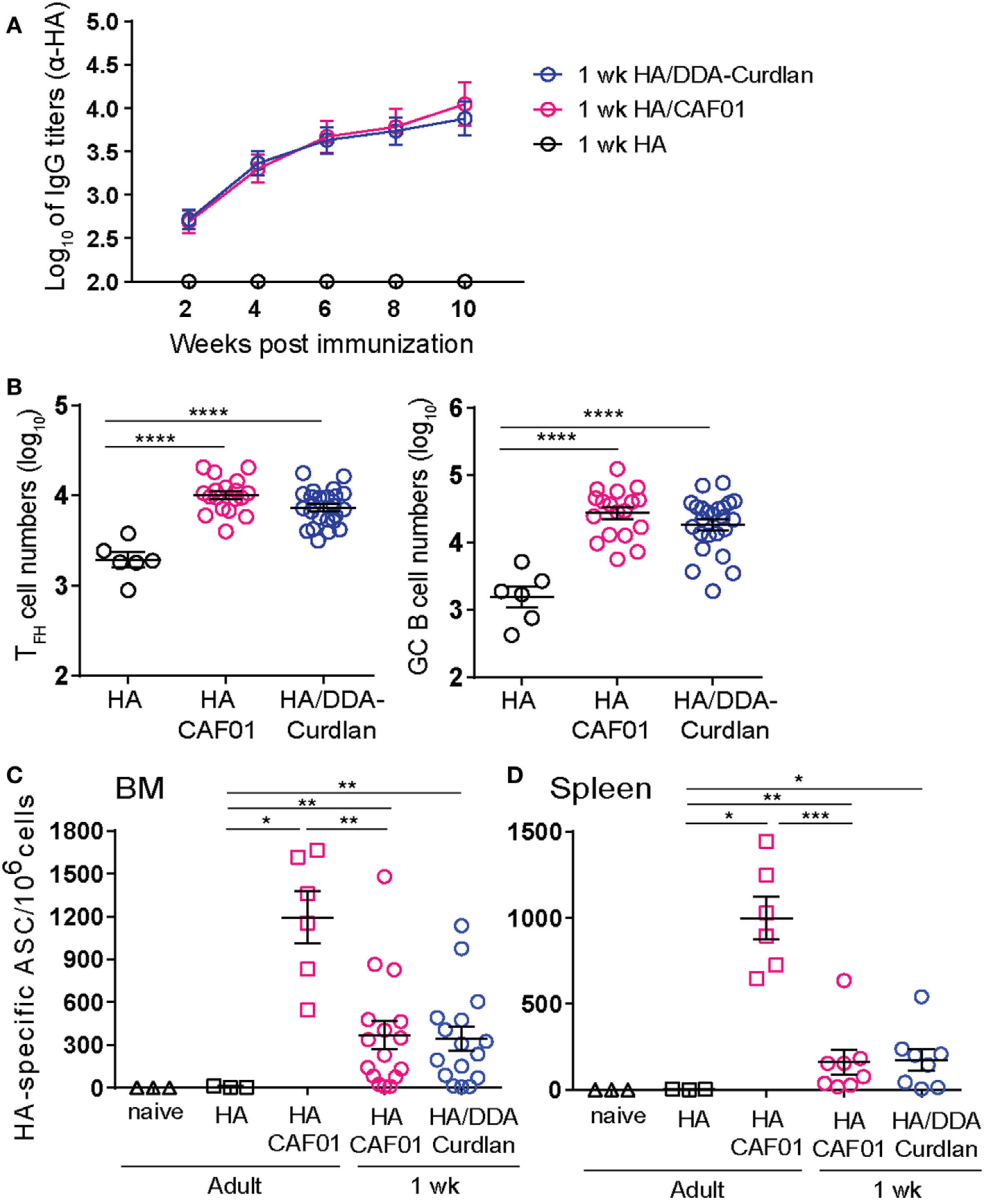
A single dose of hemagglutinin (HA)/dimethyldioctadecyl-ammonium (DDA)-Curdlan elicits primary responses similar to HA/CAF01. **(A,B)** 1-week-old mice were immunized subcutaneously (s.c.) at day 0 with HA alone, HA/CAF01, or HA/DDA-Curdlan. **(A)** Sera samples were drawn at the indicated time points to measure HA-specific IgG titers. Values represent mean logarithmic titers (log 10) of more than eight mice per group ± SEM. Both CAF01 and DDA-Curdlan significantly increased antibody titers when compared with unadjuvanted HA at all assessed time points (statistics not shown in the graph). **(B)** Graphs report the total CXCR5^high^PD-1^high^ T_FH_ cell numbers and the total GL7^+^CD95^+^ GC B cell numbers in draining lymph nodes at day 12 post-immunization. **(C,D)** 1-week-old and adult mice were immunized s.c. at day 0 as indicated and boosted 10 weeks later with HA only; 1-week post-boost antibody-secreting cells (ASC) were measured by enzyme-linked immunospot assay. The graph shows the proportions of ASCs in the bone marrow **(C)** and spleen **(D)** in the vaccinated groups and naïve mice. Data pool of three independent experiments. Mann–Whitney *U* test: *P < 0.05, **P < 0.01, ***P < 0.001, ****P < 0.0001.

We first confirmed the adjuvanticity of DDA-Curdlan in adult mice: a single dose of HA/DDA-Curdlan strongly enhanced HA-specific IgG antibodies compared with unadjuvanted HA [anti-HA IgG (log_10_) 4.4 ± 0.14 vs 2.1 ± 0.07, *P* < 0.001, 4 weeks post-prime], a similar effect to that induced by CAF01. Then, we vaccinated neonatal mice with unadjuvanted HA or formulated with either CAF01 or DDA-Curdlan. HA/DDA-Curdlan strongly increased primary HA-specific neonatal antibody responses, with no differences to HA/CAF01 (Figure 6A). Both adjuvants significantly increased HA-specific antibody titers when compared with unadjuvanted HA, at all assessed time points for up to 10 weeks post-prime. This reflected the enhancement of both T_FH_ and GC B cell responses by DDA-Curdlan, with no differences compared with CAF01 (Figure 6B).

Next, we evaluated the capacity of both formulations to induce antibody-secreting cells (ASCs) able to home into the bone marrow (BM), another hallmark of potent GC reactions which is rarely reached in early life (28). Neonatal mice were immunized s.c. at day 0 with HA/CAF01 or HA/DDA-Curdlan. Adult mice receiving HA/CAF01, HA only or naïve were included as control groups. Except for naive animals, all groups of mice were boosted 10 weeks later with HA only and ASCs were measured 1-week post-boost by ELISpot assay. High numbers of HA-specific ASCs were retrieved from the neonatal BM after vaccination with either HA/CAF01 or HA/DDA-Curdlan (Figure 6C). Neonatal responses were higher than those observed in adults immunized with HA only, but did not reach the levels observed in adults immunized with HA/CAF01. High numbers of ASCs were also observed in the spleens of immunized neonates (Figure 6D). Thus, the neonatal B cell adjuvanticity of CAF01 and DDA-Curdlan results in a potent and sustained induction of *bona fide* GC B cells, which rapidly generates high-affinity and long-lived ASCs.

## Discussion

In this study, we explored the neonatal potency of three adjuvants in clinical development given their strong B-cell promoting activity in adults, and we identified the Mincle agonist-containing adjuvant CAF01—but not the TLR-based adjuvants GLA-SE and IC31^®^—as capable of inducing *bona fide* GC responses and thus robust and prolonged primary humoral responses in murine neonates. Our major observations with CAF01 were extended to an agonist targeting a distinct CLR (Curdlan/Dectin-1), revealing the potency of CLR agonists over TLR-based adjuvants in circumventing the limitations of neonatal B cell responses to current early life vaccines.

TLR agonists are a group of arising adjuvants, some of which have already been approved for human use or are currently in clinical trials. Monophosphoryl Lipid A (MPL), a potent agonist of TLR4, is currently in use in combination with alum in vaccines against hepatitis B and papilloma virus in adults. AS01, containing MPL and the saponin QS-21, was included in the candidate RTS,S malaria vaccine in infants and children affected by HIV and improved responses to vaccination (31). TLR7/8 agonists enhanced responses to vaccination in neonatal mice and rhesus macaques (32) and their ability to activate APCs from human cord blood *in vitro* is well documented (33–37). CpG oligonucleotides, agonists of TLR9, partially circumvented the T_H_2 polarization of neonatal responses to vaccines and increased antibody responses to distinct antigens (8, 10, 38). Considering the growing evidence of adjuvant activity of TLR agonists on neonatal APCs and T cells, and the strong GC-inducing capacity of both GLA-SE and IC31^®^ adjuvants in adult mice, their weak neonatal B cell adjuvanticity was unexpected.

Reduced TLR-mediated responses have been reported in early life (39, 40) and may limit the functionality of GLA-SE and IC31^®^ in this age group. However, their lack of early life B cell adjuvanticity did not concur with T cell unresponsiveness to the TLR4 or TLR9-agonists: both HA/GLA-SE or HA/IC31^®^ increased T_FH_ cell responses compared with unadjuvanted HA, suggesting sufficient APC activation to initiate the T_FH_ cell differentiation process and induce adult-like T_FH_ cell numbers. In adults, T_FH_ cell responses directly translate into GC induction and strong antibody responses. This is not the case in neonates, indicating the existence of additional requirements for the optimal induction of GC responses by TLR agonists. In humans, adult-like TLR4- and TLR9-mediated APC/T cell responses are typically achieved during the first year of life (7, 41). Should the induction of T_FH_ cell responses not directly translate into B cell responses in humans as observed in mice, the youngest age at which HA/GLA-SE or HA/IC31^®^ might induce potent B cell responses to primary vaccination may thus not be predicted.

CAF01 includes the C-type lectin agonist TDB, which signals through Mincle and the Syk-Card9 pathway (23). A recent report showed that TDB activates human newborn DCs and greatly enhanced T_H_1 polarizing cytokine production by DCs when given in combination with a TLR7/8-ligand (42), extending previous preclinical reports of the unique efficacy of CAF01 to induce T_H_1/T_H_17 responses in murine neonates (43). In adults, the B cell adjuvanticity of CAF01 is lower than that of GLA-SE or IC31^®^ (12, 44). Its greater capacity to trigger the differentiation of neonatal B cells into *bona fide* GCs was thus unexpected. It does not merely result from its DDA-associated depot effect (29), also exhibited by IC31^®^ (15), but likely from the activation of a CLR-mediated pathway—as shown by the similar GC-promoting capacity of CAF01 and DDA-Curdlan in neonates. Curdlan was shown to enhance T_H_1 responses to a subunit TB vaccine in neonatal mice (45) and neonatal human monocyte-derived DCs readily responded to Dectin-1 and TLR7/8 agonists by producing IL-12p70 (45). Importantly, this study is the first evidence of its potent neonatal B cell adjuvanticity *in vivo*.

What may explain the higher neonatal B cell adjuvanticity of CLR- over TLR-based adjuvants?

The recognition by TLRs mainly triggers intracellular signaling cascades that result in APC maturation and the induction of inflammatory cytokines, leading to T cell activation (46, 47). In contrast, CLRs are known to perform as efficient endocytic receptors for antigen on the surface of APCs, especially on DCs where they are highly expressed (48). CLRs main function is to internalize their ligand antigens for degradation in lysosomal compartments and to enhance antigen processing and presentation by DCs and other APCs (49, 50). Efficient APC maturation in turn may provide better T cell activation. CLRs do not only function as antigen uptake receptors, they also facilitate efficient loading of antigen on MHC class I and II molecules and induce both antigen-specific CD8 and CD4 T cell responses (51, 52).

Although their APC activation capacities may differ, all tested adjuvants efficiently triggered the induction of T_FH_ cells, which is in contrast to what we observed with aluminum salt-based or MF59^®^-adjuvanted influenza vaccines in neonates (6, 10). However, the quality of T_FH_ cells elicited by CLR- vs TLR-based adjuvants might differ. T_FH_ cell functionality, hallmarked by high-expression levels of Bcl6, ICOS, and secretion of cytokines such as IL-21 and IL-4, is critical for optimally cognate T_FH_/B cell interactions in GCs (53, 54) and subject to current studies. Another hypothesis is that CLR- and TLR-based activation may essentially differ at the B cell level. Both GLA-SE and IC31^®^ induce a small number of GC B cells in neonates, although these fail to develop into *bona fide* GC structures. Efficiently activated APCs by CLR agonists may provide early GC B cells with the amount of antigen required for development and persistence of GC.

Moreover, it would be interesting to study whether CLR-based adjuvants share common mechanisms with LT-K63, the first adjuvant shown to induce early and persistent antibodies responses in neonatal mice (8). All these questions are now open for studies focusing on the relative GC B cell inducing capacity of adjuvants considered for use in early life.

Despite the strong adjuvanticity of CAF01 in neonates, GC and antibody responses did not reach the levels elicited by adjuvanted vaccines in adults. This suggests that additional neonatal limiting factors may be addressed to further improve B cell adjuvants for early life.

The identification of CAF01—a safe adjuvant currently in clinical development—as a potent neonatal adjuvant, the definition of its mode of adjuvanticity through the induction of *bona fide* GC responses and the demonstration that this property is shared by a distinct CLR agonist are major steps forwards. This paves the way to a large area of investigation to identify CLR agonist-containing adjuvants or combination-derivatives thereof, that are able to induce the most appropriate and effective responses to vaccination in early life.

## Ethics Statement

This study was carried out in accordance with the recommendations of the Geneva veterinary office and conducted under relevant Swiss and European guidelines. The protocol was approved by the Geneva veterinary office.

## Author Contributions

MV, CSE, EM, FA, DC, P-HL, BM-G, and C-AS contributed to formulation of theory and prediction. MV, CSE, P-HL, BM-G, and C-AS designed research. BM-G, MV, CSE, EM, FA performed the experiments and analyzed and/or interpreted the data. MV, CSE, BM-G, and C-AS wrote the manuscript. DC, MS, RC, AM, and PA provided reagents and critically revised the manuscript. All authors reviewed the manuscript.

## Conflict of Interest Statement

PA and DC are co-inventors on patent applications covering CAF01. As employees, DC and PA have assigned all rights to Statens Serum Institut, a Danish non-profit governmental institute. RC is an employee at the Infectious Disease Research Institute, Seattle. AM is an employee at Valneva Austria GmbH. Other authors have no conflict of interest to declare.
