# Supplementary material for: Overcoming the Neonatal Limitations of Inducing Germinal Centers through Liposome-Based Adjuvants Including C-Type Lectin Agonists Trehalose Dibehenate or Curdlan
Source: Front Immunol. 2018 Feb 28;9:381. doi: 10.3389/fimmu.2018.00381 (PMC5835515; doi:10.3389/fimmu.2018.00381)

Supplementary Material

**Overcoming the Neonatal Limitations of Inducing Germinal Centers through Liposome-based Adjuvants including C-type Lectin Agonists Trehalose Dibehenate or Curdlan**

Maria Vono, Christiane Sigrid Eberhardt, Elodie Mohr, Floriane Auderset, Dennis Christensen, Mirco Schmolke, Rhea Coler, Andreas Meinke, Peter Andersen, Paul-Henri Lambert, Beatris Mastelic-Gavillet and Claire-Anne Siegrist^*^

*** Correspondence:** Claire-Anne Siegrist: Claire-Anne.Siegrist@unige.ch

# Supplementary Figures

**
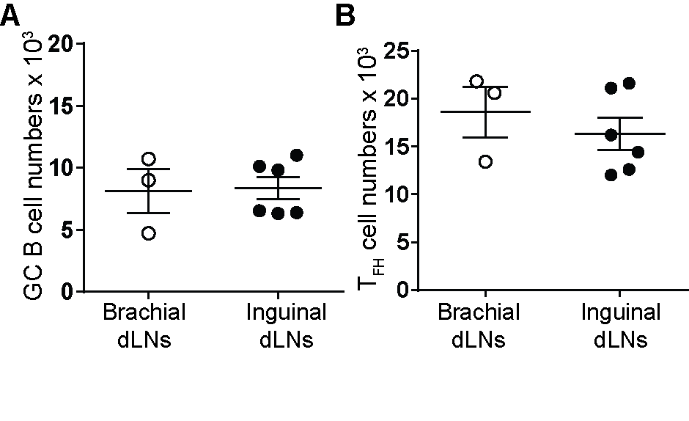
**

**Supplementary Figure 1.** **Comparison of germinal center (GC) B cell and T follicular helper (T_FH_) cell responses induced by HA/GLA-SE in brachial versus inguinal draining LNs.** Neonatal CB6F1 mice were immunized s.c. with HA/GLA-SE in the scruff of the neck or at the base of the tail and the brachial or inguinal draining LNs were collected respectively at day 10 upon immunization. Total numbers of B220^+^ GL7^+^ CD95^+^ GC B cells **(A)** and CD4^+^ CXCR5^high^PD-1^high^ T_FH_ cells **(B)** were measured by flow cytometry. Results are expressed as mean ± SEM.

**
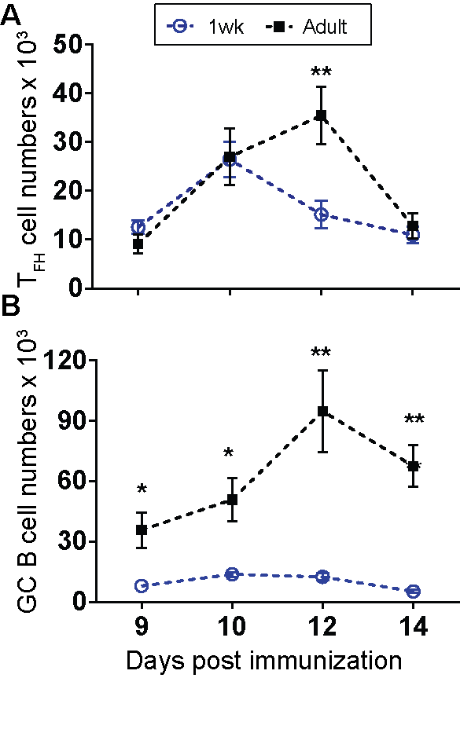
**

**Supplementary Figure 2. Kinetics of T_FH_ and GC B cell responses induced by HA/GLA-SE in draining LNs.** Neonatal and adult CB6F1 mice were immunized at day 0 s.c. in the scruff of the neck with HA/GLA-SE. The brachial draining LNs were collected on days 9, 10, 12, and 14 after immunization, and total numbers of CD4^+^ CXCR5^high^PD-1^high^ T_FH_ cells **(A)** and B220^+^ GL7^+^ CD95^+^ GC B cells **(B)** were measured by flow cytometry. Results are expressed as mean ± SEM. Statistical analysis were performed using the Mann-Whitney U test: **P* < 0.05, ***P* < 0.001.


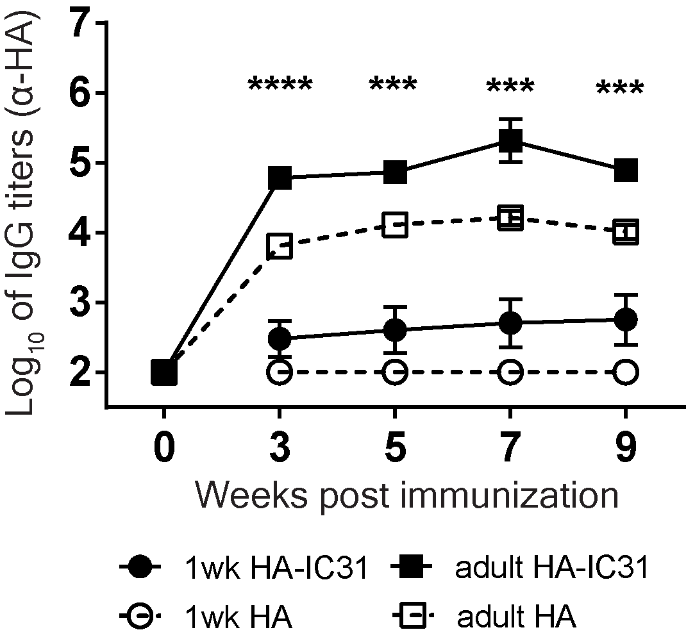


**Supplementary Figure 3. IC31^®^-induced antibodies to HA in neonatal mice remain low over time**. Neonatal or adult CB6F1 mice were immunized s.c. at day 0 with HA alone or formulated in combination with IC31^®^. Sera were drawn at the indicated time points post immunization and total HA-specific IgG antibody titers were measured by ELISA. Values represent mean logarithmic titers (log_10_) ± SEM. Adjuvanted groups were compared to the corresponding non-adjuvanted HA groups using the Mann-Whitney U test: ****P* < 0.0001, *****P* < 0.00001. (*) refer to adult mice; the adjuvant did not significantly increase HA-specific IgG titers in neonates when compared to HA alone.

**
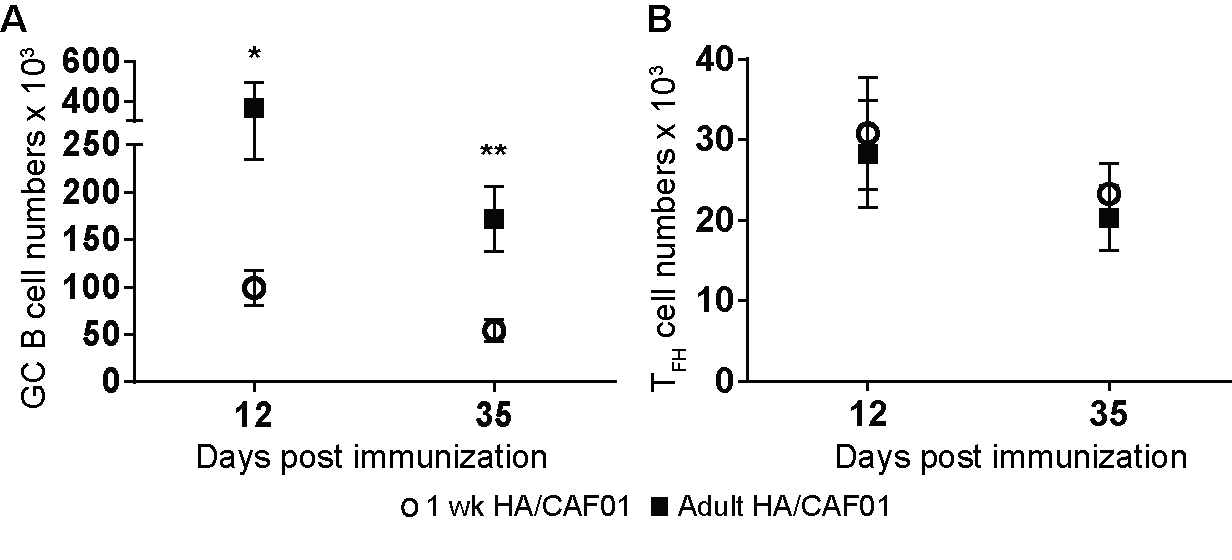
**

**Supplementary Figure 4.** **Germinal Center B cell and T_FH_ cell responses induced by a single dose of HA/CAF01 persist over time for at least 35 days post immunization in both neonates and adults.** Naïve CB6F1 neonatal and adult mice were immunized s.c. at day 0 with HA/CAF01 (5-8 mice per group). Draining LNs were harvested 12 or 35 days post immunization. The numbers of B220^+^ GL7^+^CD95^+^ GC B cells (**A**) and CD4^+^ CXCR5^high^PD-1^high^ T_FH_ cells (**B**) were measured by flow cytometry. Results are expressed as mean ± SEM. Statistical analysis were performed using the Mann-Whitney U test: **P* < 0.05, ***P* < 0.001.

**Supplementary Images**

The original microscopy images included in Figure 3C of the manuscript are shown below.

These are representative sections of adult and neonatal draining lymph nodes showing

immunohistochemical staining for IgD (green), peanut agglutinin (PNA;red) and CD4 (purple) for all indicated conditions.

- 1wk HA (image from Mirax)


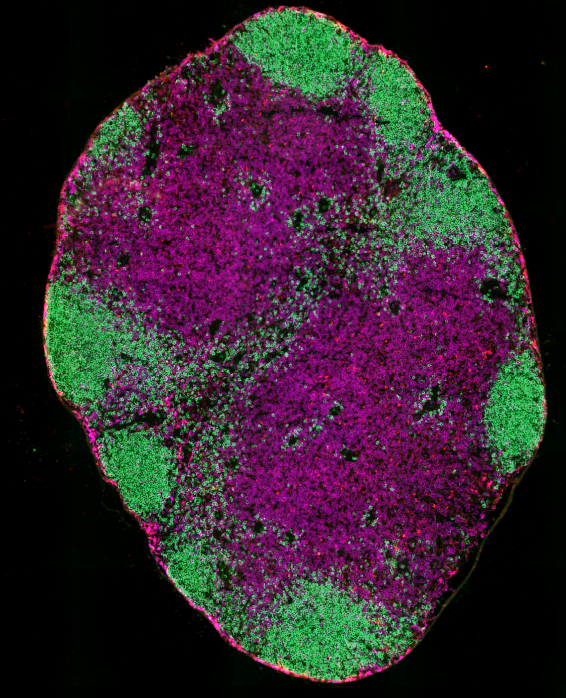


- 1wk HA/CAF01 (image from Mirax)


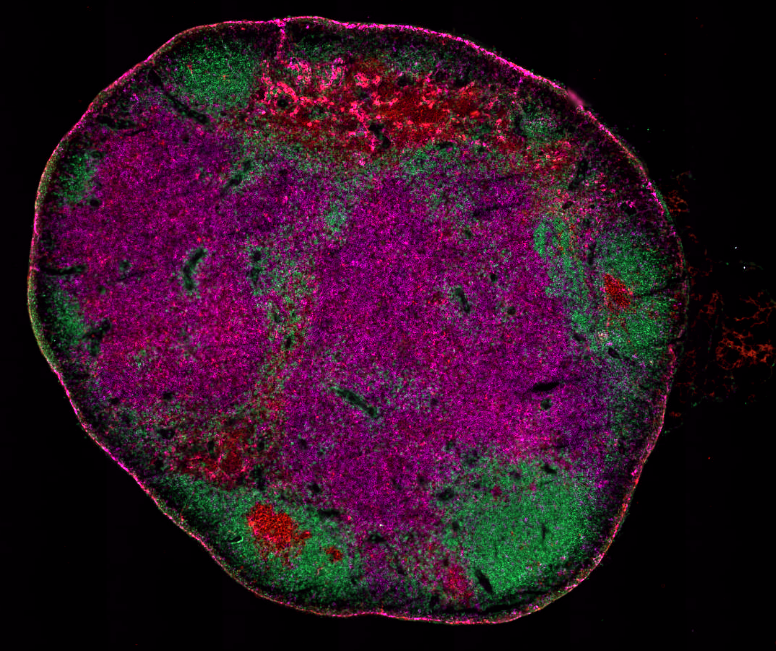


- Adult HA (image from Mirax)


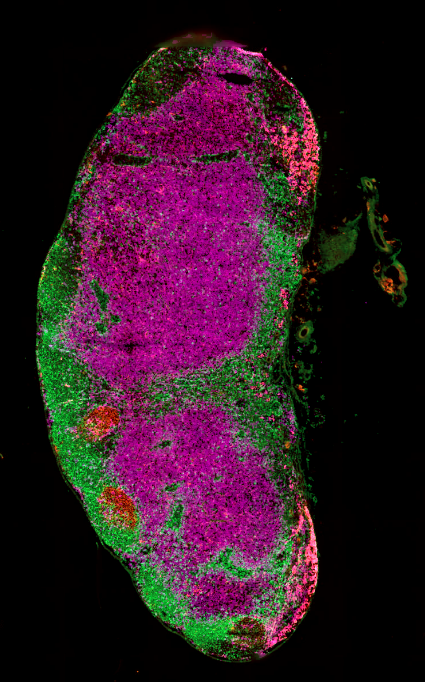


- Adult HA/CAF01 (image from Mirax)


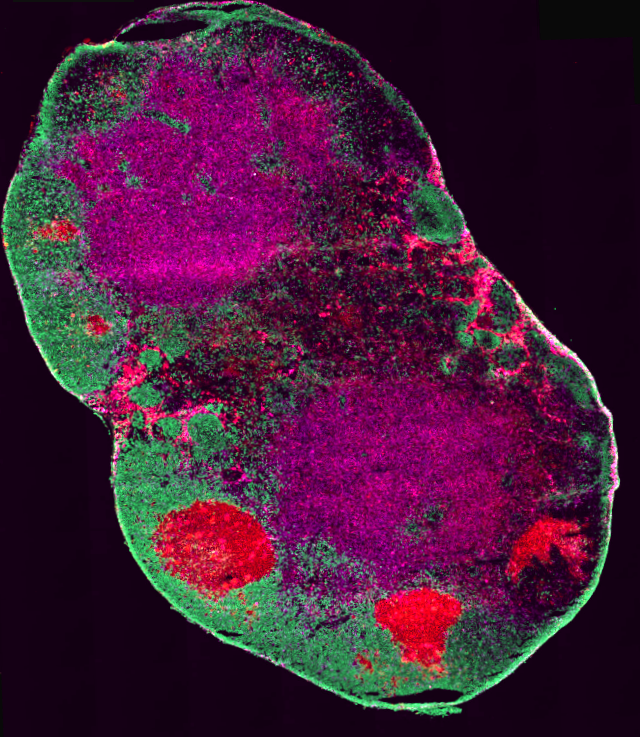


- 1wk HA/CAF01 (image from Confocal microscopy)


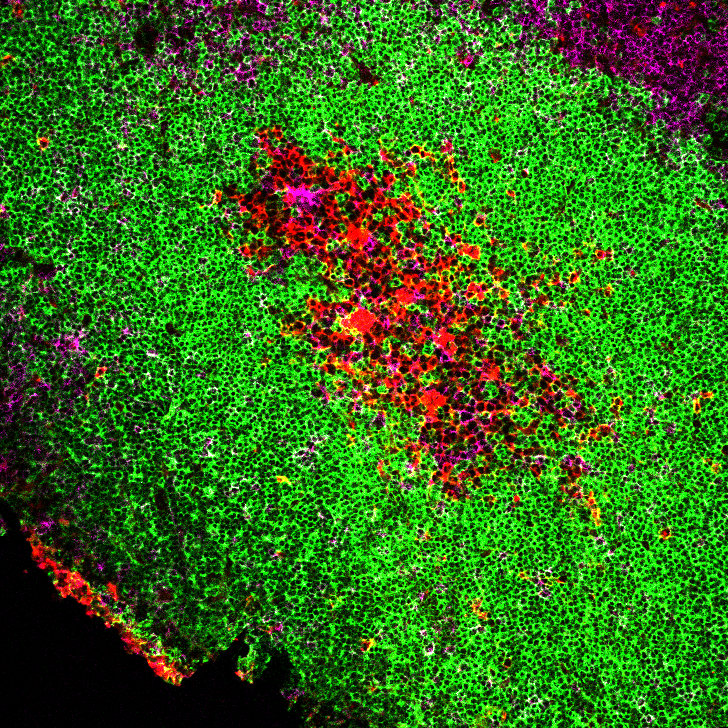


- 1wk HA/IC31 (image from Confocal microscopy)


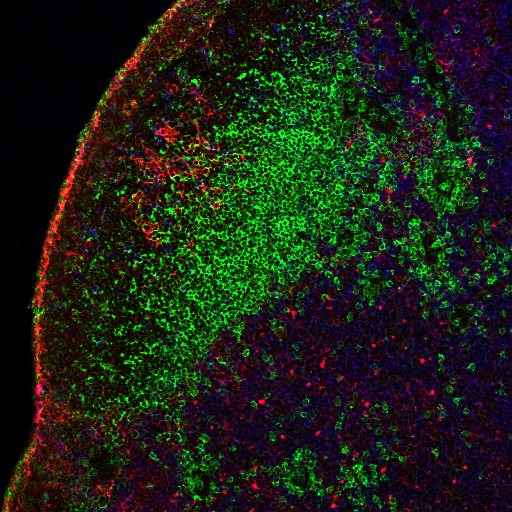


- 1wk HA/GLA-SE (image from Confocal microscopy)


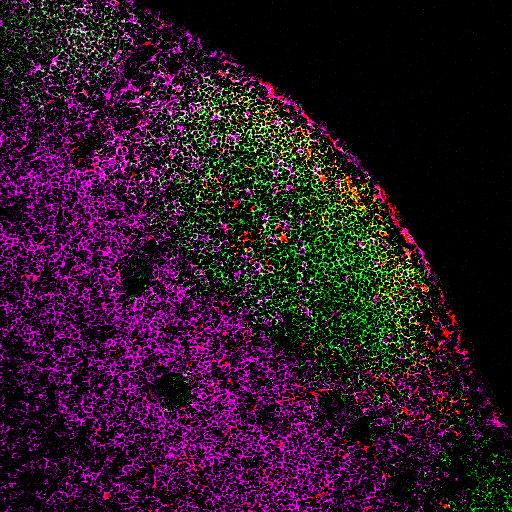


- Adult HA/CAF01 (image from Confocal microscopy)


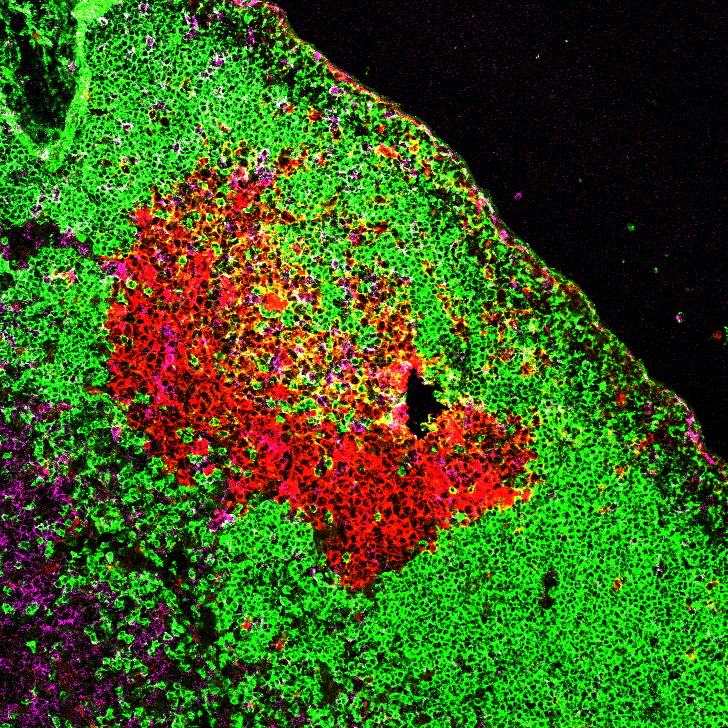


- Adult HA/IC31 (image from Confocal microscopy)


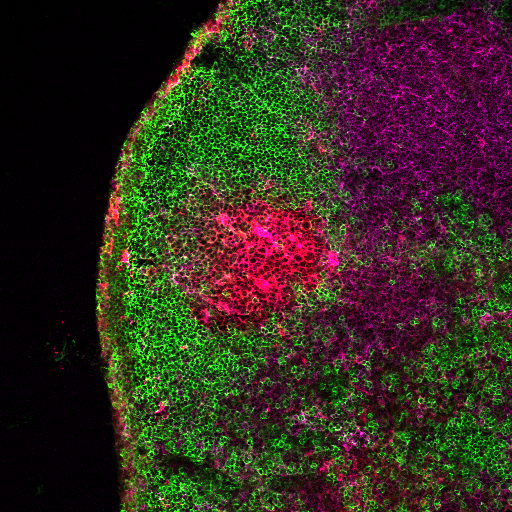


- Adult HA/GLA-SE (image from Confocal microscopy)


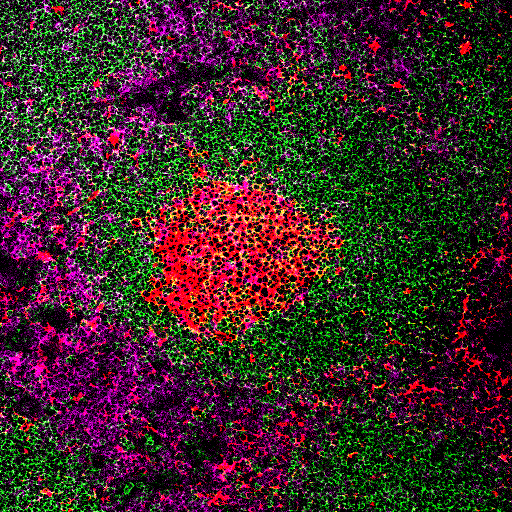

Supplement: Supplementary file 1 [file data_sheet_1.docx]
